# Maintaining close canopy cover prevents the invasion of *Pinus radiata*: Basic ecology to manage native forest invasibility

**DOI:** 10.1371/journal.pone.0210849

**Published:** 2019-05-24

**Authors:** Persy Gómez, Maureen Murúa, José San Martín, Estefany Goncalves, Ramiro O. Bustamante

**Affiliations:** 1 Jardín Botánico, Universidad de Talca, Talca, Chile; 2 Centro GEMA- Genómica, Ecología y Medio Ambiente, Universidad Mayor, Santiago, Chile; 3 Instituto de Biología Vegetal y Biotecnología, Universidad de Talca, Talca, Chile; 4 Departamento de Ciencias Ecológicas, Facultad de Ciencias, Universidad de Chile, Santiago, Chile; 5 Instituto de Ecología y Biodiversidad (IEB), Facultad de Ciencias, Universidad de Chile, Santiago, Chile; Technical University in Zvolen, SLOVAKIA

## Abstract

Pine invasion is a global threat that is occurring in native forests of diverse regions of the world. This process is arising in a scenario of rapid forest deforestation and degradation. Therefore, elucidate which forests attributes explain invasibility is a central issue in forest ecology. The Coastal Maulino forest is an endemic forest of central Chile, which has suffered a large history of disturbance, being replaced by large extensions of *Pinus radiata* plantations. This land transformation conveys high rates of pines invasion into native remnants. In this study we examined to what extent structural features of forest patches explains invasibility of this forest-type. Within eight forest fragments, we sampled 162 plots (10 x 10 m^2^ each). We quantified seedling pine density and related these estimates with tree cover, litter depth, PAR radiation, and diversity of the resident community. Our results indicate that canopy cover was the most important variable to determine seedling pine density within forest fragments. Our investigation highlights the importance to conserve the forests cover to reduce significantly their invasibility. This action can be effective even if we cannot avoid pine plantations in the region as a source of a massive seed dispersal to forests with well conserved canopy.

## Introduction

Annually, millions of hectares are deforested and fragmented worldwide, transforming native forests into disturbed habitats often used for agriculture and forestry practices [1]. This land transformation produces a cascade of ecological effects, being one of them high rates of species invasion into the native remnants [2–5].

The invasibility of native forests, i.e. their capacity to resist invasion, is dependent on different forest attributes: Firstly, as more diverse are native forests, they are more resistant to invasion; as community is saturated, resources are depleted, competition is intense and no more species can establish [6,7]. However, if plant resources (such as light or nutrients) increase in time, competition can be reduced, therefore resources can be utilized by exotic plants, thus increasing forest invasibility [8]. More recently, species relatedness has emerged as other factor to define invasibility. If within a recipient community there are species which are close relatives with the potential invader, they can outcompete invaders [9,10]. Another factor that increases forest invasibility is disturbance regime. For instance, a reduction of forest cover by human activities, increases light availability, then, luminous microsites become available for the establishment of shade-intolerant exotic plant which outcompete native shade-tolerant plants establishing in the forest [11–16]. Thus, forest invasibility is a dynamic attribute that can be modulated by resource supply, disturbance regimes and phylogenetic structure of recipient communities [8]. These factors acting in synergy, determine a complex scenario to predict forest invasibility.

Trees of the family Pinaceae, are highly invasive in the southern hemisphere [17]. Because of their importance as commercial forestry crops and ornamental use, pines have been widely transported by people around the world [18]. This species has attracted the attention of ecologists due to the tremendous areal extent of invasion as well as the ecological, economic and sociological impacts [18,19]. Diverse traits has been proposed to predict pine invasion: species with small seeds, short length of juvenile period, and short interval between large seed crops constitute good predictors of pine invasiveness [20]: From these studies, the most invasive pines are, in decreasing importance: *Pinus contorta*, *P*. *halapensis*, *P*. *pinaster*, and *P*. *radiata* [20].

In general, forest are not invaded by pines because most of them are shade-intolerants and they cannot establish under the forest canopies [21,22]. *Pseudtsuga menziesii*, is one of the few shade-tolerant conifer tree (at least during juvenile phases) and regarded highly invasive in temperate forests [23,24]. In a scenario of growing disturbance (deforestation and fragmentation) [1], the forest environment can change dramatically, thus increasing its invasibility.

*Pinus radiata* is a shade–intolerant tree species original from California (USA) [25]. Regarded the fourth most invasive pine in the Southern Hemisphere [20], this species has impacted biodiversity significantly [17,26,27]. This invasive success should be is concordant with: (i) fire, which promotes regeneration due to serotony, i.e. an adaptation exhibited by some seed plants, in which seed release and germination occurs in response to an environmental trigger, in the case of pines, fire [15]; (ii) deforestation and habitat fragmentation which reduce native cover promoting invasion [25,28,29]; and (iii) the diversity of resident plants which could affect invasion by competition, particularly if there are native relatives (or not) in the recipient forest [30]. In this study, we examined the invasibility of the Coastal Maulino forest, an endemic forest of Central Chile [31]. Specifically, we assessed whether structural attributes and micro-environmental factors affect *Pinus radiata* regeneration. Given the levels of forest fragmentation and the huge amount of *P*. *radiata plantations* surrounding remnant fragments [32], the Coastal Maulino forest is ideal to test the hypothesis that disturbance (expressed in changes of forest cover) as well as local native species diversity will affect the invasion of *P*. *radiata*. Specifically, we predict a) a negative relationship between pine regeneration (assessed as the seedling density) and forest cover; b) a negative relationship between pine regeneration and native species diversity; c) a significant interaction between forest cover and native species diversity.

## Methods

**Fig 1:**
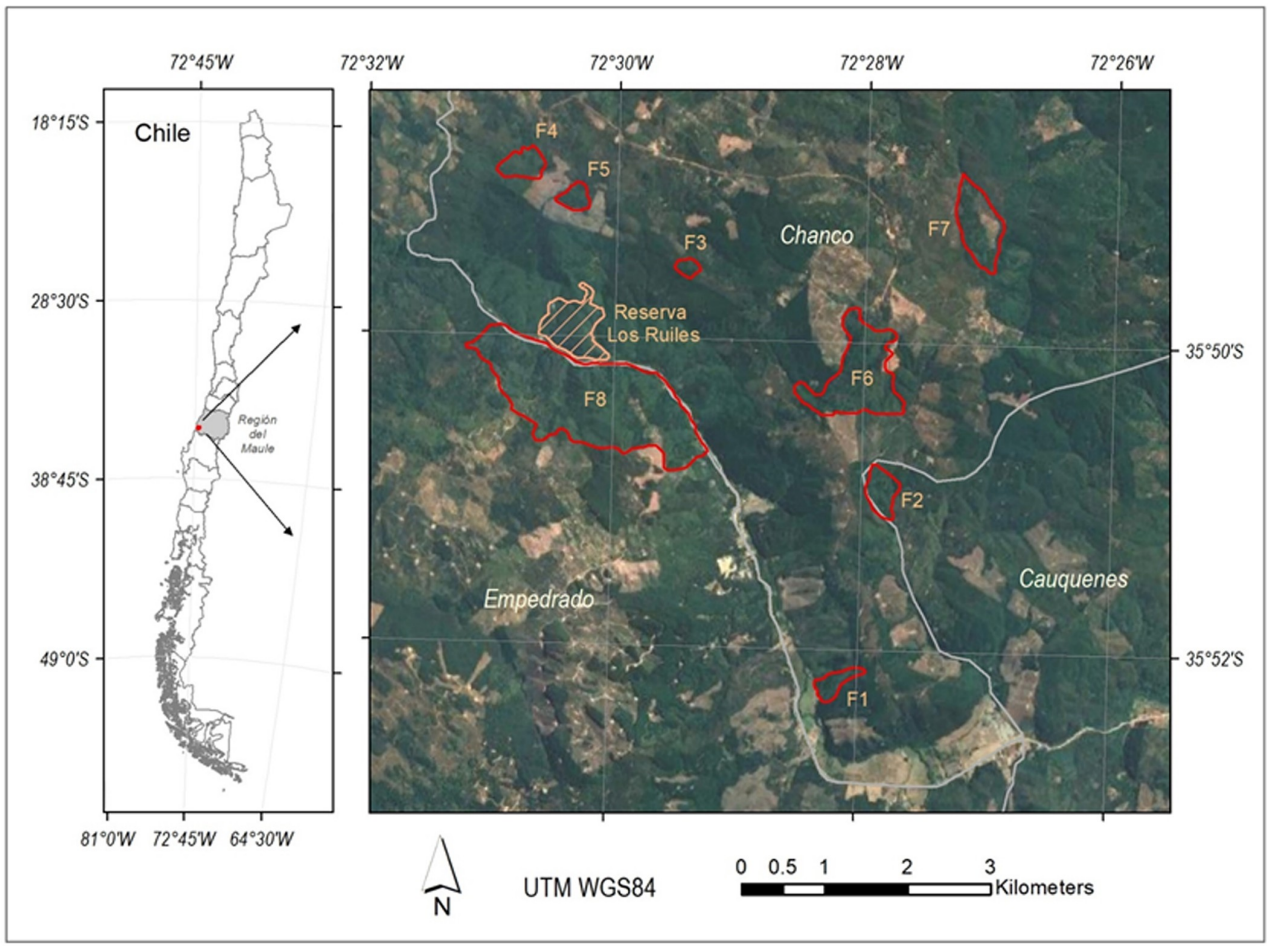
Spatial distribution of Coastal Maulino forest fragments, Cauquenes, Maule Region, Chile. Red contours show eight fragments of native forest; the remaining colors indicate pine plantations in different development phases. In achurate: National Reserve Los Ruiles.

The Coastal Maulino forest is located in the coastal range between 35° and 37° latitude S [31]. It is an endemic ecosystem which has declined significantly the last decades [33]. For instance, ca. 67% of the original forest was replaced by *Pinus radiata* D. Don between 1975 and 2000 [34]. Currently, forest patches are embedded in a matrix of pine plantations [32]. Pine invasion into forest fragments occurred at least from 1945 and currently, there exists approx. 10% of reproductive individuals inside forest [35]. During the last decades the intensity and extent of human-induced fires have increased significantly, reducing even more the remnant native forest [34]. The study area was located at Cauquenes, Maule Region (-72.35°; -35.97°). Topography is heterogeneous with plains, gently slopes and creeks. The climate is Mediterranean-type with mean annual temperature of 18°C and mean annual precipitation of 709 mm, concentrated mainly in the winter season [36,37]. The landscape is highly anthropogenic presenting a mosaic of native forest fragments surrounded by *Pinus radiata* plantations. Dominant native tree are *Nothofagus glauca* associated with *Persea lingue* and *Gevuina avellana* in more humid habitats and with *Nothofagus obliqua* and *Nothofagus alessandrii* in drier habitats [31]. Evidence indicates that *P*. *radiata* is invading actively these forest [11,38]. Our research focused on eight forest fragments dominated by *N*. *glauca*, which ranged from 3 to 152 ha, summing a total of 332 ha; the areal extent of the sampled zone was approx. 60 km^2^ (Fig 1). This study was conducted in private lands of

Forestal Mininco SA. (www.cmpc.cl), with their corresponding permissions.

We randomly set 162 sampling plots of 0.01 ha (properly geo-referenced), inside fragments; the number of plots per fragments was set up proportionally to their area (see S1 Table). We attempted that the ratio *fragment area/total area of plots* ranged from 1 to 2, with the exception to the case of the largest one (ratio 3.46, see Fragment 8 in S1 Table. Within each plot, we quantified seedling pine abundance (response variable), species diversity and richness of native plant species, forest cover, litter width and PAR radiation. A seedling pine was defined as a young plant whose height was below or equal to 1 m. For species diversity and species richness within plots, we identified plants at species level, we recorded their abundance (in numbers), its relative abundance and finally, the Shannon-Weiner index, excluding explicitly *P*. *radiata*. Forest cover was photographed using a camera SONY 10.1 mega pixel, disposed on tripod, 1.20 m height over the ground. To estimate the canopy cover (in percentage), the photographs were analyzed using the SIGMA (SCAN) software. We cannot discard some contribution of adult pines (10 m height or more) to the forest cover; however, it should be negligible given the low relative abundance of this species (Average = 3%; Standard Error = 0,9%). Litter depth (cm) was assessed using a rule 30 cm-long in 5 randomly selected points within sampling plots. PAR radiation was measured using a radiometer LI-COR, Model LI-250 Light Meter USA; these measures were taken at the ground level. We tested for spatial dependence among plots, conducting a Mantel test, correlating the matrix of the geographic distance between pairs of plots with the matrix of the pine seedling density differences between pairs of plots, using the plotrix package of R [39].

Within the region of study (approx. 60 km^2^, Fig 1), we found eight forest fragments, accessible for research with remarkable differences in size, isolation degree and shape (Fig 1, S1 Table). Given the extremely deforestation and fragmentation process suffered by the Coastal Maulino Forest in the past [32,34], it is almost impossible to selected fragments which share similar environmental conditions; then we cannot avoid to include in the analysis a suite of undesirable sources of variation, which deserve to be controlled (Fig 1). In order to do it, we considered each fragment as a block and sampling plots nested within fragments. We conducted a GLMM analysis with randomized block design (with negative binomial distribution) to assess the effect of species diversity, forest cover, litter width and PAR radiation on pine seedling density (response variable). Before the statistical analysis, we performed a Principal Component Analysis (PCA), to reduce multi-collinearity among independent variables [40,41]; then, the GLMM analysis was run using the fragments as randomized blocks and the two first PC as fixed effects. The reason to consider fragments as blocks because we exclude unknown fragment-specific effects, which makes estimates for fixed effects more transferable.

We modeled the occurrence probability of pine seedling, P(O), along a forest cover gradient using the hierarchical logistic Huisman-Olff-Fresco (HOF) regression models [42]. This analysis is regarded an efficient method to describe species responses along ecological gradients [43,44]. The HOF models were conducted using “eHOF” package in R [39]. This analysis provides a set of five models for selection. The best model was selected by AIC criteria and using 1000 bootstrapping permutations. To get the goodness of fit of the model, a *pseudo* R^2^ was also estimated [45]. As the dataset is more than less balanced (*P*. *radiata* present in 100 of 162 plots), we decided to select a threshold value P(O) = 0.5. For all statistical analysis, we used R package version 3.5.3 (Core Team 2019).

## Results

**Fig 2:**
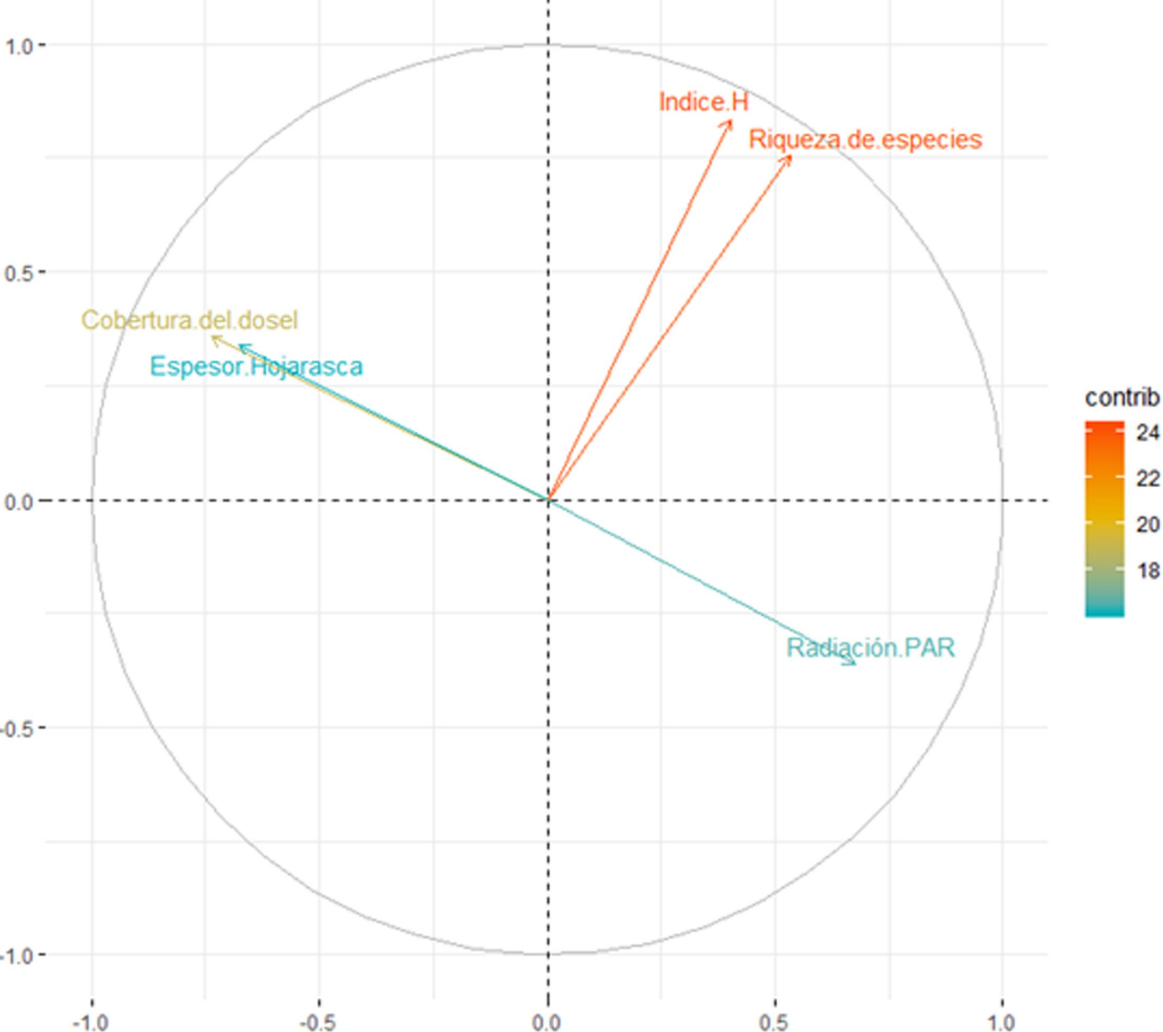
Principal Component Analysis (PCA) with variables that explain independent variables related with seedling density of *Pinus radiata*, Maule Region, Chile. PC1 explained 38,2% of total variation while PC2, accounted 33% of the variance.

Mantel test indicated that there is no spatial dependence among pairs of plots (r = 0.015; P = 0.31), i.e. the geographical distance between pairs of plots did not affect differences in seedling abundance. From PCA, the PC1 accounted 38% of the variance (eigenvalue = 1.90), and correlated negatively with tree cover (r = -0.53), and litter depth (r = - 0.49) and positively with PAR radiation (r = 0.48) (Fig 2). PC1 is an axe that represents light availability, being negative values with low light availability and positive values an increase of light availability. On the other hand, PC2 accounted 33% of the variance (eigenvalue = 1.64), and was correlated positively with Shannon-Wiener index (r = 0.65) and species richness (r = 0.59) (Fig 2). Thus, PC2 is an axe that represents native species diversity, being negative values with low diversity while positive values with high diversity.

**Fig 3:**
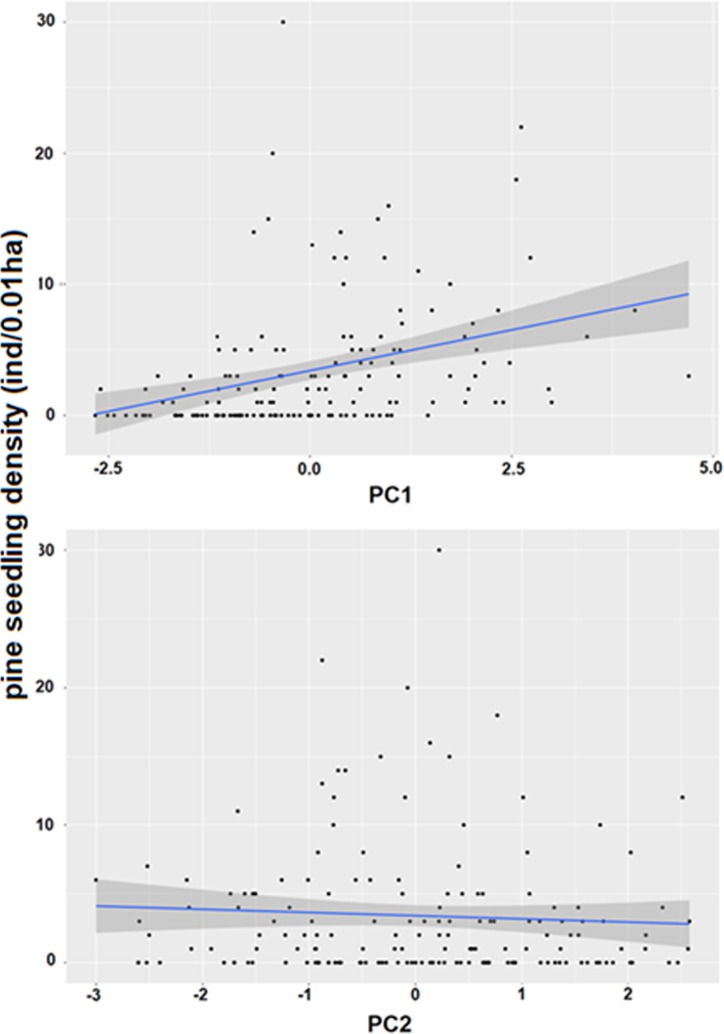
Relationship between pine seedling density and the two first principal components. (A) Relationship between pine seedling density and PC1; (B) Relationship between pine seedling density and PC2. For both figures, the fitted model was linear.

From GLMM analysis, we detected an important block effect (0.157 ± 0.40; average ± std. dev.), however this between-fragment variability was not enough to detect that pine seedling density was positively and significantly affected by PC1 (Fig 3A, Table 1); no significant effects were detected neither for PC2 (Fig 3B, Table 1) nor for the interaction PC1 x PC2 (Table 1).

**Table 1:** General linear mixed model (GLMM) with negative binomial distribution, to assess the relationship between PC1 and PC2 and pine seedling abundance of *Pinus radiata* in fragmented forests, Maule Region, Central Chile.

SOURCE OF VARIATION	DF	Z value	P
PC1	1	5.04	**<< 0.001**
PC2	1	-1.93	0.06
PCI x PC2	1	0.93	0.36

**Fig 4:**
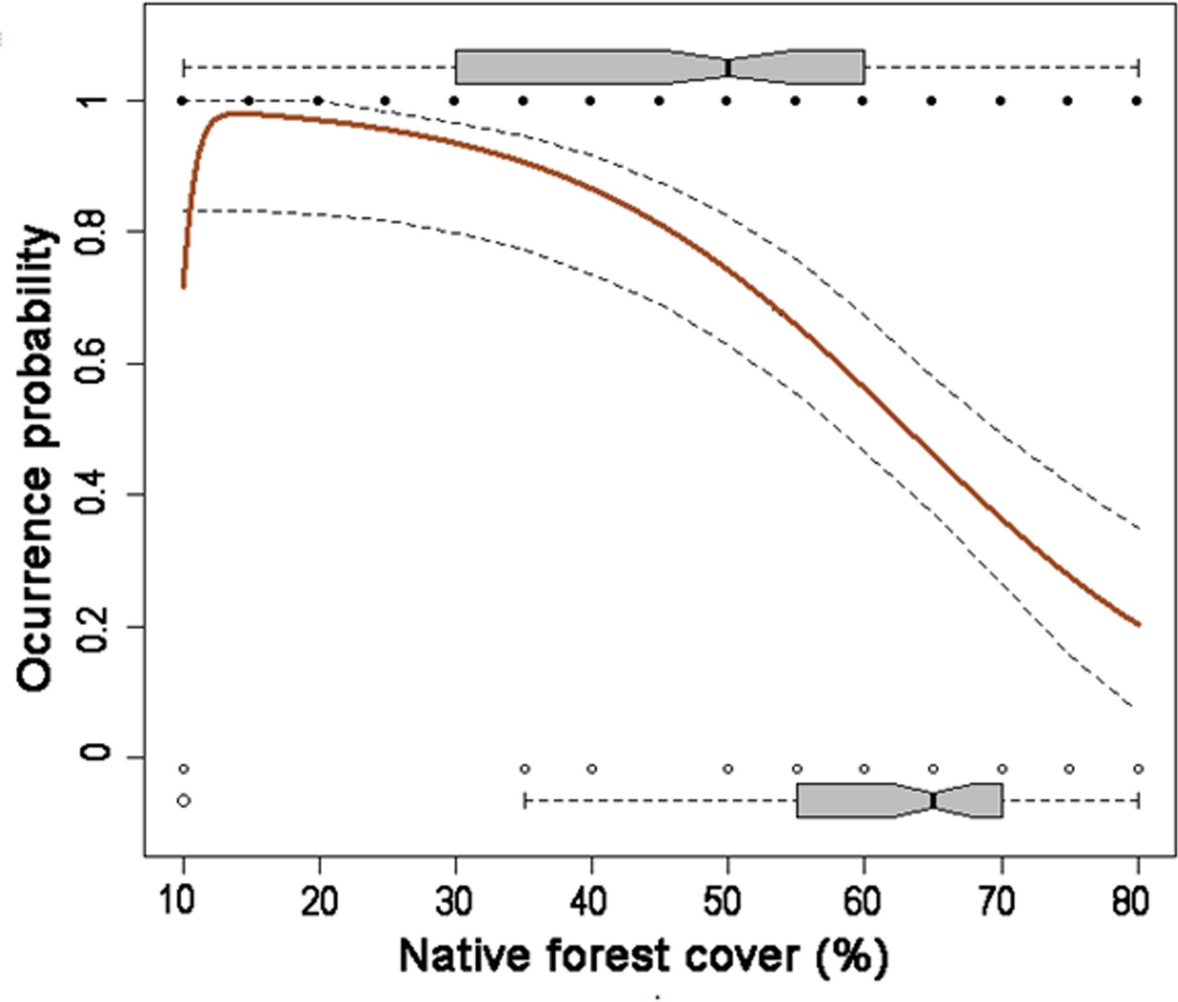
Logistic regression analysis relating forest canopy cover and occurrence probability P(O) of *Pinus radiata* seedlings in the Coastal Maulino forest. Red line describes the model; dashed lines are 95% confidence intervals. Black dots are presences; white dots are absences. For more clarity and practical use, we decided to relate P(O) with values of native forest cover directly. The analysis was conducted with n = 162 plots.

The HOF model-type V (Fig 4) was the best model based on its lowest deviance, AIC value and bootstrapped analysis (58%) (see S2 Table). The optimum values of P(O) occurred at a forest cover of 14%. The threshold value under which P(O) was equal to lower than 0.5 was 63%. Note that at cover values, lower than 40%, P(O) attained almost 1.

## Discussion

In this study, we have tested the hypothesis that forest invasibility can be predicted by the interplay between canopy cover (a proxy of disturbance) and resident species diversity (a proxy of competition intensity) [8]. Our results indicate that canopy cover is sufficient to determine forest invasibility. As this variable is a proxy of light availability, it is the ultimate abiotic driver influencing pine regeneration. We did not detect direct effects of the native plant community on pine regeneration, suggesting no biotic resistance (competition) of the resident community. This result is of major importance because indicates that there are no biotic filters for the colonization and establishment of this invasive tree.

The forest canopy is the first biotic filter to arrest pine invasion. From a neutral model, we expect a massive invasion only due to propagule pressure with independence of any other ecological factor: as more propagules arrive to the forest, more invasion is expected [46,47]. Forest fragments are surrounded by a matrix of pine plantations and are subject to a massive seed rain. *Pinus radiata* trees become reproductive at five years of age [25], the rotation period of plantations is about 25 years [48]. This means that each individual will produce seed for a period of 20 years before cutting. This seed rain, transported by winds, have the possibility to disperse to the interior of the largest forest fragments [38]; notwithstanding, pine establishment resulted limited if the canopy cover was high.

The reduction of pine recruitment due to forest canopy has been also reported in other *Pinus* species such as *Pinus contorta*, *Pinus halepensis* and *Pinus brutia* [25,49,50]; given the importance of this variable, there have been some attempts to classify ecosystem invasibility based on canopy cover: forests with a very closed canopy are the most resistant, followed by open woodlands, shrublands, and finally grasslands [51].

One intriguing result was the collinearity of litter and light availability; in some cases, litter can be a barrier for small seedlings. However, in our study this effect can be negligible given that pine seedling was sufficiently tall to overcome presumable potential negative effects of litter depth.

The Coastal Maulino forest is a deciduous forest-type [31], i.e. most of native trees lose their leaves during autumn-winter (like the dominant tree *Nothofagus glauca*); this fall occurs in relative synchrony with seed dispersal season of *Pinus radiata*, thus providing a temporal window with open conditions suitable for a successful seedling recruitment [38]. The high invasiveness of *P*. *radiata* results, in part, from its capacity to germinate (but not recruit) under a wide range of conditions [17,38,49]. If germinated seeds can survive to the next leaf fall period, when light availability increases again, then pine invasion to the interior of the forest remnants will be a matter of time [52].

From logistic regression analysis, a reduction of the canopy cover dramatically increases the seedling density of *P*. *radiata*, with a threshold easy to achieve due to human disturbances. In consequence, any disruption of canopy cover will provide a suitable scenario for *Pinus* invasion. As human activities related to logging or recurrent fires reduce canopy cover, there are new opportunities to *P*. *radiata* invasion and probably other invasive species with similar regeneration requirements (e.g. *Teline monpessulana*, [35]). During the last years, massive fires have occurred across Central Chile [53]. Surely, all of these fires were originated by people with devastating ecological effects [54,55]. From the point of view of *Pinus radiata*, this scenario is optimal for invasion because the native cover is reduced dramatically as it has been documented by previous studies in other regions of the world [56,57].

Despite our study included an intense sampling effort (*n =* 162 plots) across 8 forest fragments, thus encompassing a wide spatial extent (see Fig 1), there is a notable variation of data (Fig 3) which suggests the existence of other factors, not included in our study, which may also explain pine regeneration. Several studies have tested and proven the effect of landscape variables, as for example, proximity to nearby towns and pine plantations, the forest edge extension, the size and the area/perimeter ratio on the distribution and abundance of alien plants [58,59]. Future studies ought to focus on a more inclusive approach encompassing landscape features in statistical models to allow a better understanding of pine invasion and improve our predictive capacity to assess invasibility of the last remnants of the Maulino forest.

### The future of the coastal Maulino forest

Successional studies in the Coastal Maulino forest, using Markovian matrices, predict successional changes from a deciduous–type forest (current) to a more sclerophylous-type with a dominance of species such as *Cryptocarya alba*, *Lithraea caustica*, mid- and late successional tree species with perennial leaves [60]. These successional studies did not include *P*. *radiata* as a novel component of the forest. Up to date, the contribution of pines to the canopy results negligible (0.3%). However, this picture can change in the future. The inclusion of pines in successional models should reinforce the predicted successional path because pines produce a dense and persistent canopy, favoring to shade-tolerants [31], specially, if we know that the regeneration of the native tree *Cryptocarya alba* (a shade-tolerant tree) is not limited with *P*. *radiata* litter [61].

The future of the remnants of the Maulino forest is uncertain; the recent mega-fire events which occurred in 2017, have modified severely its structure, thus giving rise to ideal conditions for the invasion of *Pinus radiata*. In fact, we have observed a massive recruitment of this exotic tree. The protection of this unique native forest is an urgent task for private land owners, and public authorities. We hope that our study can help to this valuable task.

## Conclusion

Our results, besides to corroborate the hypothesis that canopy cover is the best predictor to arrest pine invasion, as it has documented in other studies, provide basic information to conduct simple conservation actions in the Coastal Maulino forest. To preserve this valuable ecosystem and the containing biodiversity, it is mandatory to conserve the canopy cover beyond the detected threshold of 63%. These solely actions will be highly effective even if there exists a massive seed rain that arrives each year from the surrounding pine plantations, as they are unable to recruit under well conserved native forests [38]. These criteria can be properly used in other regions of the world where native forests are in permanent risk to be invaded by pines.

## Supporting information

S1 Table Number of sampled plots within eight forest fragments in Coastal Maulino forest, Cauquenes, Central Chile. (DOCX)

S2 Table Deviance, AICC values and bootstrap tests for the selection of the best model from hierarchical logistic Huisman-Olff-Fresco (HOF) regression models (Huisman et al. 1993). (DOCX)

S3 Table Summary of original data collected in sampling plots. Latitude and longitude are in UTM.
(XLSX)
