# Correction: Maintaining close canopy cover prevents the invasion of *Pinus radiata*: Basic ecology to manage native forest invasibility

**DOI:** 10.1371/journal.pone.0219328

**Published:** 2019-06-28

**Authors:** Persy Gómez, Maureen Murúa, José San Martín, Estefany Goncalves, Ramiro O. Bustamante

There are errors in the caption and variable names for Fig 2, “Principal Component Analysis (PCA) with variables that explain independent variables related with seedling density of *Pinus radiata*, Maule Region, Chile.” Please see the complete, correct Fig 2 here.

**Fig 2:**
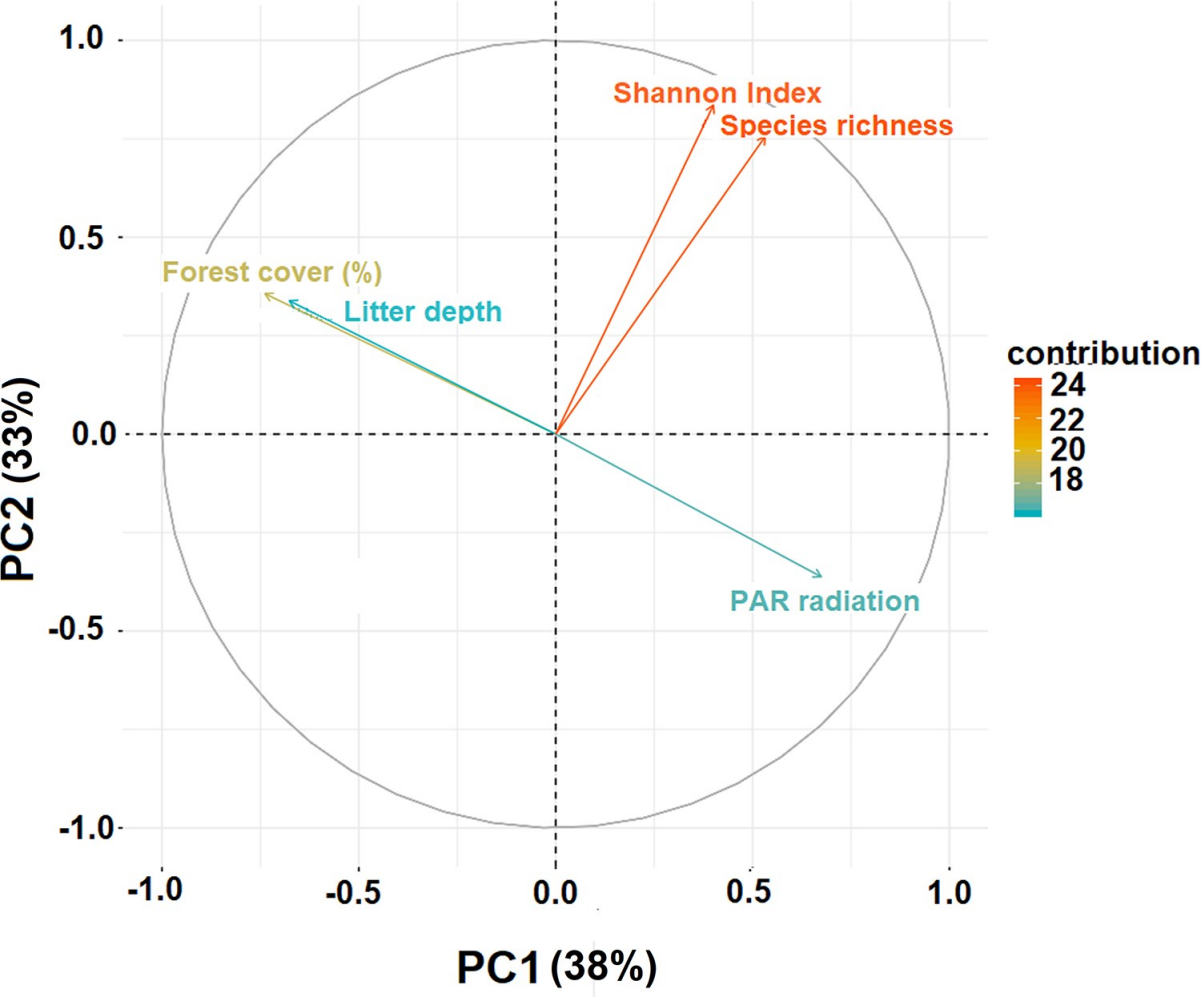
Principal Component Analysis (PCA) with variables that explain independent variables related with seedling density of *Pinus radiata*, Maule Region, Chile. PC1 explained 38% of total variation while PC2, accounted 33% of the variance.
