# Supplementary material for: Maintaining close canopy cover prevents the invasion of Pinus radiata: Basic ecology to manage native forest invasibility
Source: PLoS One. 2019 May 24;14(5):e0210849. doi: 10.1371/journal.pone.0210849 (PMC6534307; doi:10.1371/journal.pone.0210849)
Supplement: S1 Table — (DOCX) [file pone.0210849.s001.docx]

| Fragment Forest | Fragment size (ha) | Plot Number (100 m^2^ each) |
| --- | --- | --- |
| 1 | 11 | 11 |
| 2 | 19 | 15 |
| 3 | 3 | 3 |
| 4 | 20 | 16 |
| 5 | 7 | 7 |
| 6 | 70 | 35 |
| 7 | 53 | 30 |
| 8 | 152 | 45 |
| TOTAL AREA | 332 | 16200 m^2^ |
