# Supplementary material for: Maintaining close canopy cover prevents the invasion of Pinus radiata: Basic ecology to manage native forest invasibility
Source: PLoS One. 2019 May 24;14(5):e0210849. doi: 10.1371/journal.pone.0210849 (PMC6534307; doi:10.1371/journal.pone.0210849)
Supplement: S2 Table — (DOCX) [file pone.0210849.s002.docx]

| **Model** | **Deviance** | **AICC** | **Bootstrapped**  **(probability of the best model)** |
| --- | --- | --- | --- |
| I | 215.58 | 217.61 | 0 |
| II | 179.117 | 183.19 | 0.26 |
| III | 176.286 | 182.44 | 0.06 |
| IV | 175.119 | 181.27 | 0.08 |
| **V** | **172.09** | **180.34** | **0.56** |
| VI | 175.12 | 183.37 | 0.01 |
| VII | 174.72 | 185.10 | 0.03 |
